# Nursing care for older patients with pressure ulcers: A qualitative study

**DOI:** 10.1002/nop2.474

**Published:** 2020-03-10

**Authors:** Christina Louise Lindhardt, Sanne Have Beck, Jesper Ryg

**Affiliations:** ^1^ Department of Geriatric Medicine Odense University Hospital Odense Denmark; ^2^ Department of Clinical Institute University of Southern Denmark Odense Denmark; ^3^ University College Absalon Sorø Denmark

**Keywords:** nurse's experience, older patients, pressure ulcers, qualitative research, thematic analysis

## Abstract

**Aim:**

To explore the experience and perception of pressure ulcers in a group of nurses caring for older patients.

**Design:**

A qualitative study based on interviews with (*N* = 6) nurses working with older patients.

**Method:**

A qualitative approach was applied using thematic analysis influenced by Braun and Clarke.

**Results:**

The findings comprised one main theme “Prevention of pressure ulcers is important” and four sub‐themes “Nursing resources on the ward,” “Basic nursing skills—lift the duvet,” “Introduction of new nurses on the ward—bedside teaching” and “Missing articulation of pressure ulcers.” Bedside teaching and experienced nurses may create a culture on the ward where basic nursing skills and observations are articulated.

## 1 INTRODUCTION

Pressure ulcers are prevalent in hospitalized patients ranging from 5% to 32% depending on the patients in focus (National Institute for Health & Care Excellence, 2014). Risk factors include increasing age, decreased mobility, multi‐morbidity and poor nutrition (European Pressure Ulcer Advisory Panel, 2014). Patients with these risk factors are often admitted to a geriatric ward when hospitalized (Agrawal et al., 2019). Pressure ulcers are defined as areas of localized damage to the skin and underlying tissue usually over a bony prominence, as a result of pressure or pressure in combination with shear. They cause pain, lead to immobility, delay recovery and have an impact on health‐related quality of life (European Pressure Ulcer Advisory Panel, 2014; Lavalleè, Dumville, & Cullum, 2018). Furthermore, hospital‐acquired complications, such as pressure ulcers, cost more than other kinds of inpatient complexity (Bail, Draper, Berry, Karmel, & Goss, 2018; National Institute for Health & Care Excellence, 2014). Therefore, the prevention of pressure ulcers is a statistically significant healthcare issue nationally and internationally (European Pressure Ulcer Advisory Panel, 2018; National Institute for Health & Care Excellence, 2014). However, attention is needed to explore new approaches used for prevention, treatment and care of pressure ulcers, and to ensure practice is based on the best available evidence (Kottner et al., 2019; National Institute for Health & Care Excellence, 2014; National Pressure Ulcer Advisory Panel, 2014).

Pressure ulcers might be avoidable, and prevention is often seen as a nursing concern (Odierna & Zeleznik, 2003). Different tools can be used to focus on a structured risk assessment of all patients admitted to the hospital (Sving, Fredriksson, Gunningberg, & Mamhidir, 2017) with reassessments whenever the patients' conditions change (Sving et al., 2017). A study from Sving et al. found that technology towards preventing pressure ulcer, such as pressure‐reducing mattresses, reduced the use of repositioning planning (Sving, Idvall, Hogberg, & Gunningberg, 2014).

In a Danish context, at Odense University Hospital, preventing pressure ulcer became part of the patient safety programme initiated in 2012 (Department of Patient Safety, 2015–2018). The programme established an internal organization with the purpose of assessing the numbers and reasons of pressure ulcers and producing a guideline for preventing pressure ulcer. The study indicated that the staff had little knowledge about pressure ulcers and treatment of these.

## 2 AIM

The aim of this study was to explore the experience and perception of preventing pressure ulcer for geriatric nurses when caring for older patients in the daily clinic.

## 3 METHODS

A qualitative descriptive method, including a thematic analysis based on six semi‐structured individual interviews, was employed. The qualitative approach made it possible to explore the nurse's points of view and stay close to the data that were generated from the interviews. A qualitative descriptive approach as thematic analysis can be used when a straightforward phenomenon is being sought as it is the case in this study (Sandelowski, 2010).

The thematic analysis is a direct description of an experience. In the analytical process and in the presentation of the data, the researcher remains focused on the data that were generated. It is a description of the participants' experience in a language similar to that of the participants (Braun & Clarke, 2006). The qualitative interview was semi‐structured, allowing modification of the interview during the process to ensure focus on areas not yet explored. The analyses of the themes that emerge depend on the perceptions, inclinations, sensitivities and sensibilities of the researcher (Polit, 2008).

### 3.1 Participants and setting

A total of six nurses were interviewed. The nurses were recruited through a gatekeeper at the geriatric department of Odense University Hospital. Nurses were included if they had been working in the geriatric department for at least two months. The nurses had a median (range) age of 43 (24–59) years, an employment in a geriatric ward of 4 years (3 months–10 years) and between 3 months and 34 years' work experience—in total.

### 3.2 Study procedure

The interview guide was tested by pre‐interviews in nurses not included in the actual study, which resulted in corrections of the language and content. Following informed consent, qualitative interviews were conducted and digitally recorded. Interviews lasted approximately 30 min each and took place at the work place. The data were collected by an experienced qualitative researcher.

### 3.3 Thematic analysis

A thematic analysis proposed by Braun and Clarke was used (Braun & Clarke, 2006). All interviews were transcribed verbatim and analysed. To ensure the placement of a code in a theme, data consistency was searched for and codes that did not fit were contemplated and placed elsewhere. All quotations behind each of the codes were carefully read and patterns were sought.

Braun and Clarke state that in the last step the themes and sub‐themes should be related to the research question and to the literature (Braun & Clarke, 2006). This is done to safeguard validity of the qualitative description and credibility of the thematic analysis. The interviewer had a nursing background similar to the nurses interviewed. In this study, the consistency and validity of the data was raised by confirming the findings through discussions with the co‐authors of this article. The dissimilar background of the co‐authors should minimize systematic errors and contribute to protect correct coding of the themes. Consistency was ensured by ongoing critical reviews of the coding, paying attention to the context and writing a logbook with critic considerations during the process (Braun & Clarke, 2006).

In the first step, the text was read and re‐read by two researchers to ensure correctness and to exclude typing errors. In addition, the researchers debated the transcribed content. In the second step, a record of the first impressions and the perceived similarities and differences was made. In the third step, the data were systematically divided into meaningful codes. In the fourth step, these initial codes were noted and re‐viewed, and thus, codes became visible. In the fifth step, the coded data were advanced into a thematic map‐making where the researchers considered the adjustment of themes and sub‐themes. In the sixth and final step, each theme was analytically refined and related to the literature and evident definitions were made for each theme and sub‐themes.

### 3.4 Ethical considerations

Based on the study design, approval from an ethical committee was not necessary, due to Danish legislation. All participants gave informed consent. According to Danish law, personal data were saved according to good clinical practice and confidentiality.

## 4 RESULTS

Generally, all interviewed nurses conveyed that pressure ulcers are a topic that needs attention when caring for old patients in a geriatric ward. However, the nurses anticipated challenges. The analysis of the interviews resulted in the identification of one overall theme and four sub‐themes (Table 1).

**Table 1:** The overall theme and the four sub‐themes

Overall theme
Prevention of pressure ulcers is important in the care of geriatric patients
Sub‐themes
Nursing Resources' on the ward
Basic nursing skills—lift the duvet
Introduction of new nurses on the ward—bedside teaching
Missing articulation of “pressure ulcers”

### 4.1 Overall theme: Prevention of pressure ulcers is important in the care of geriatric patients

All the nurses interviewed stated that the topic of pressure ulcers was of essential importance in nursing care, in particular when caring for geriatric patients. It was expressed that skin care and observations hereof is considered as one of the fundamental nursing tasks in nursing geriatric patients.

During the interviews, four sub‐themes emerged and all sub‐themes were chosen according to how the nurses explained their experiences with perception and perception of pressure ulcers: (a) Nursing resources on the ward, (b) Basic nursing skills—lift the duvet, (c) Introduction of new nurses on the ward—bedside teaching and (d) Missing articulation of “pressure ulcers.”

### 4.2 Sub‐theme 1: Nursing resources on the ward

Several of the nurses expressed concern about the nursing resources on the ward. They feel the daily workload on the ward prevents that clinical guidelines and theories of pressure ulcers become part of the nursing care without sufficient time to engage in this issue. They express that they often have to make compromises on their basic nursing care and observations of the patients due to this.

Other factors mentioned were the factual work frame, the physical locations, the computer systems and the healthcare professionals with specific knowledge on pressure ulcers availability on daily basis in the ward. Also, the intensity of care to the individual patients influenced the daily treatment of patients with pressure ulcers:

… we are a bit challenged on resources. …the time I use on administrative things, it is time away from being and observing the patient, so I have to depend on the observations made by my SSA (healthcare Assistant colleagues) (Nurse 3)

…we do have too few resources to task (Nurse 6).

### 4.3 Sub‐theme 2: Basic nursing skills—lift the duvet

It was expressed by the nurses that basic nursing skills are of utmost importance when taking care of geriatric patients in particular. The transfer from novice in nursing to expert becomes challenging as the geriatric patients with comorbidities need individual care and planning:

…we have to perform a ‘primary pressure ulcer screening’ and then we have made ‘daily inspection’ that is the two things we have… (Nurse 5)

It is noted that there were differences in how the individual nurses were trained as nurse students. Some of the nurses described how they had gone through their whole training at the faculty for nursing studies without ever having seen a pressure ulcer. Others said that they had met the patients with pressure ulcers in the district nursing in the municipality. Subsequently, their starting point when being employed in the geriatric ward was not equal:

… in our education we learn about pressure ulcer and also in the practical parts of the education. In my first job I had a mentor who said, “you have to lift each patient's duvet, every day” (Nurse 3)

### 4.4 Sub‐theme 3: Introduction of new nurses on the ward—bedside teaching

All the nurses mentioned introduction of new nurses on the ward as crucial when getting a feel for nursing geriatric patients in general and in particular when observing and detecting pressure ulcers. Bedside teaching was mentioned more than once as a method of introducing the nurses to the special care of geriatric patients. One nurse described that when she was new to the geriatric field she was asked by her colleagues:

have you seen this…, have you tried that

But when it came to preventing pressure ulcer, she explained:

We (nurses) are not good at saying; “come with me and see” when it is in relation to regular/basic care, when the patients' skin is starting to turn red, or the patient is starting to develop a pressure ulcer (Nurse 3)

Having previous experience from other wards (e.g. plastic surgical and geronto‐psychiatry) was mentioned by one of the nurses as being of great value when observing pressure ulcers. It was mentioned that to prevent pressure ulcers all nurses ought to have some kind of rotational visits to other specialized wards to gain this specific expertise.

Further, it was stated that disturbing a colleague was only acceptable if there was great concern for the patient:

I don't think you bring a colleague if the patient only has a bit of redness or starting of a pressure (Nurse 1)

When challenged on this statement, the nurse suggested that it could be a cultural thing on the ward not to disturb each other when they were busy.

### 4.5 Sub‐theme 4: Missing articulation of “pressure ulcers”

Some of the nurses said they had the experience that the nursing group on the ward did not speak of pressure ulcers in general. Further, that the daily management of the ward and caring for the patients did not encourage time for a deeper conversation on the topic of pressure ulcers. The lack of terminology describing pressure ulcers and speaking about observation of the patients in danger of or having pressure ulcers was also a concern.

Only one of the six nurses described a focus on the prevention of pressure ulcers:

…you have to help the patient change position, physiologically, you know, it [the body] is not built to be placed in the same position for more than two hours… it doesn't have to be a lot, you just have to help them change position to move the pressure (Nurse 4)

Another nurse emphasized:

… if I help a colleague I might suggest that she elevates the headboard … or say I think it would be a good idea to help the patient to lay on the side, to avoid pressure (Nurse 3)

This nurse reflected on the importance of helping the patient changing position. However, she did not go more into detail about it.

When talking about pressure ulcers, the nurses used general terms:

they can look so different, it is complexed (Nurse 6)

Another nurse said that she looked at and touched the area and observed for infection to conclude the state of the pressure ulcer (Nurse 6).

When raising the question, nurse 4 replied:

…there are some routines in screening for pressure ulcers and on behalf of the result we choose the mattress for the bed. And then we do some other things, those we don't give so much thought but they are also part of preventing pressure ulcer, such as helping the patient getting out of bed (Nurse 4)

The nurse further said:

I look how the patient is lying in the bed and look for the colour on the skin (Nurse 4)

The nurses described preventing and caring for patients with pressure ulcer in general terms, not specific or detailed about their observations and not about their actions or reflections of caring for a patient with pressure ulcer.

## 5 DISCUSSION

In summary, this study indicates that most nurses interviewed agreed that the prevention of pressure ulcers in the care of geriatric patients was crucial. Our findings also indicate that geriatric nurses find it challenging to navigate between preventing/observing pressure ulcers and the daily tasks on the ward. An example could be the limitation of time allocated to the individual patient's care, documenting of the data throughout the work shift and making sure information is being forwarded to the patient, relatives and healthcare colleagues.

Although most of the participants said they had been in the geriatric care for a longer period of time, it became evident that there was a gap in their knowledge of pressure ulcers that made them uncertain about when to act. This became an obstacle in their daily care as they did not wish to disturb their colleague unnecessarily.

The geriatric wards often consist a mixture of healthcare professionals who have been for longer or shorter periods on the ward (Kada, Nygaard, Mukesh, & Geitung, 2009). On the geriatric ward, the daily set‐up commences of a system consisting of several nurses in charge. However, the health assistants document and forward their observations to the nurses, who subsequently are responsible for the contact to the primary sector (district nurses). A transfer of theoretical knowledge into clinical nursing may enhance the prevention of pressure ulcers and bridge the gap when information between healthcare professionals in general is forwarded (Duhamel, 2010).

A difference in the perception of observing the geriatric patient according to the level of training in nursing has been suggested (Kada et al., 2009). This might influence the observations of pressure ulcers being reported from nursing auxiliary to the nurse. Further observations may be lost if reported hours later when forwarded during shifts. The local electronic patient file system holds an opportunity to report about pressure ulcers separately. Although the system allows the nurses to describe their observation, it lacks the space for a summary of important information and following tasks, for example pressure ulcers.

Despite nurses are presented for skin anatomy and skincare during their training (College UU, 2018; Lillebaelt UC, 2019), nurse students are not necessarily confronted with the prevention and caring of patients with pressure ulcers during their clinical training. This makes the introduction of new nurses on the ward of vital importance as previous experience may not include observing, treating and caring for pressure ulcers.

Whereas nurses do have a specific language about their observations, our study showed that the specific day‐to‐day articulation about pressure ulcers is missing out. There is a tendency in health care that the written observations and reflections diminish when using a computer system where ticking off is an easy option (Iedema et al., 2011). However, sparse literature exists on this topic. Sving et al (Sving et al., 2017) states that by changing the training it creates an awareness and understanding of working with pressure ulcer prevention: from treating to preventing. Furthermore, they emphasize that a changed understanding enables changes actions (Sving et al., 2017). The individual nurse's own care and engagement increase the quality of detecting and caring for pressure ulcers. Having a joined view on pressure ulcer prevention, easy access to pressure‐reducing equipment and external and internal peer support are important factors for changed practices (Sving et al., 2017). Bedside teaching support, feedback and discussions on current results increase the awareness of needed improvements (Sving et al., 2017).

It is mentioned in the literature that nurses' attitudes could be crucial for changing understanding and working more preventatively (Sving et al., 2017). It is recommended that whatever strategies the nurses chose to use; it needs to be tailored to fit the context and set up on the geriatric ward.

In our study, it became evident that there was a lack of knowledge and active vocabulary when addressing topics on pressure ulcers. Similar studies from other areas of nursing care (Miller, Neelon, Kish‐Smith, Whitney, & Burant, 2017) indicate a gap in knowledge related to pressure ulcers' prevention and that education and bedside training is important. This is supported by a cross‐sectional survey (Fulbrook, Lawrence, & Miles, 2019) mentioning a focused education strategy when preventing and managing pressure ulcers.

Healthcare technology has reached the area of nursing care as a popular tool (Frich & Olesen, 2015). Nevertheless, there are potential downsides in using healthcare technology in nursing as it may reduce the observation of basic patient needs and enhance recoding vital signs such as temperature, pulse and respiration without being face‐to‐face with the patient. In nursing, this may be seen as a reduction of using the trained eye and intuition (Martinsen, 2017). There is a tendency in nursing that the younger generation of nurses welcomes the technology in health care but need more time to get a routine about the basic nursing skills. This challenges nursing and in particular the geriatric patient as they are more dependent as patients and thus need trained eye of the nurses.

Despite communication being vital in health care at all levels, our findings indicate that awareness of communication is needed. This may enhance the articulation of pressure ulcers. In this way, the feedback from the bedside observations and staff room discussions among the nurses could be brought into the open (Sving et al., 2017).

In geriatrics, healthcare professionals must in particular aim at delivering high‐quality care to their patients. Here, pressure ulcers are one of the crucial factors. According to Hommel et al. (Hommel, Gunningberg, Idvall, & Baath, 2017), it may be easier to achieve this in smaller hospitals with a smaller flow of patients. Several of the nurses indicated that neglect of preventing pressure ulcers occurs in the daily clinic due to time pressure and lack of bedside teaching. A recent review states the importance of reporting “missed care” to increase the quality and establish guidelines to prevent similar situations (Recio‐Saucedo et al., 2018).

Management of healthcare professionals in geriatrics is important to embrace some of the challenges raised by the participants in this study. Similar studies mention that in particular the nurse managers' attitude and engagement are important to enable the healthcare professionals to work actively with pressure ulcer prevention (Hommel et al., 2017). It is crucial for caring for geriatric patients with pressure ulcers that there are experienced nurses involved to encourage and support their peers in the daily care and nursing. Further, the nurses should strive for a preventative mindset where topics such as pressure ulcers should be discussed in the nursing group (Hommel et al., 2017). Regular feedback on the quality of care and on those occasions allocates time for discussion and reflection.

The main strength of this study is the use of a validated and scientifically robust qualitative method to explore nursing care for patients with pressure ulcers in a geriatric ward. This could not have been achieved using a quantitative study. A limitation is the relative small number of interviewed participants. However, we managed to generate a density of data and reaching data saturation. Another limitation was the inclusion of geriatric nurses only. However, our aim was to explore the issue of pressure ulcers in this specific nursing population. Therefore, our findings might not be transferable to other nursing settings. A larger study including nurses from different geriatric hospital settings is recommended.

## 6 CONCLUSION

Our study showed that the nurses working in a geriatric ward conveyed pressure ulcers as a topic that needs attention when caring for older frail geriatric patients. A thorough introduction to the ward is essential in order for particularly the recently qualified nurses to navigate between the administrative tasks, nursing the patient and learning from more experienced colleagues. An increased awareness of using the same terminology when caring and observing patients with pressure ulcers may enhance the level of preventing and detection of patients with pressure ulcers on the ward. An opportunity could be bedside teaching/observing for novice and more experienced nurses on the ward. Finally, creating a culture on the ward where basic nursing skills and observations are articulated in the daily clinic might further increase awareness.

## AUTHOR CONTRIBUTIONS

CL Lindhardt is the principal investigator and conducted the interviews and holding the responsibility for the writing process and submission. SH Bech has been involved in the recruiting of the participant, the analysis and writing of the article. J Ryg has been part of the recruiting process and gaining access to the geriatric department. He has contributed to the writing process in all steps along the way.

## ETHICAL APPROVAL

Research Ethics Committee approval was sought at the Region of Southern Denmark's research unit. The response was that this kind of qualitative research does not require approval. All data regulations had been kept according to Danish law.
